# Patrick Manson: A Physician Pioneer in Parasitology Research

**DOI:** 10.7759/cureus.71975

**Published:** 2024-10-20

**Authors:** Binoth Navilson, Joy Bazroy

**Affiliations:** 1 Preventive and Social Medicine, Pondicherry Institute of Medical Sciences, Puducherry, IND

**Keywords:** clinical parasitology, infectious and tropical diseases, preventive and social medicine, public health education, vector-borne diseases

## Abstract

Patrick Manson, a physician-scientist who worked in China between 1866 and 1889, made significant discoveries about the transmission of tropical infectious diseases. He identified that many such diseases require a vector, unique to warmer climates, to facilitate human-to-human transmission. Manson demonstrated that microfilariae have nocturnal periodicity in the blood of patients with elephantiasis. He showed that these microfilariae undergo transformation on ingestion by mosquitoes, which act as vectors to complete their life cycle. By observing operculated eggs in the sputum samples, Manson discovered that the lung fluke caused endemic hemoptysis. He hypothesized that hatched miracidia utilize freshwater crustaceans, such as snails, as intermediate hosts, which is crucial for the life cycle of many trematodes. His groundbreaking work on vectors laid the foundation for modern vector control strategies, now essential to the World Health Organization's efforts to eliminate or control diseases like lymphatic filariasis, malaria, and dracunculiasis. Before leaving China, Manson contributed significantly to medical education and services, establishing the Alice Memorial Hospital, the Hong Kong College of Medicine for Chinese (which later became the University of Hong Kong), and the Hong Kong Medical Society. He was also the founder of the London School of Hygiene and Tropical Medicine. Each discovery not only advanced scientific understanding but also had profound implications for public health strategies in tropical regions.

## Introduction and background

Sir Patrick Manson was a pioneering British parasitologist and is recognized as the father of tropical medicine. He was the first to demonstrate, between 1877 and 1879, that mosquitoes could serve as hosts for developing parasites, specifically the *Wuchereria* *bancrofti* worm, which causes filariasis in humans. He was a pioneer in arthropod-borne disease transmission dynamics. Arthropod-borne diseases primarily refer to viral illnesses which are transmitted through arthropod (insect) vectors like mosquitoes, ticks, and mites. This article aims to provide an overview of Manson's remarkable life and scientific career, assess the importance of his groundbreaking discoveries, and situate these findings within the modern understanding of arthropod-borne diseases.

## Review

Early life and education

**Figure 1:**
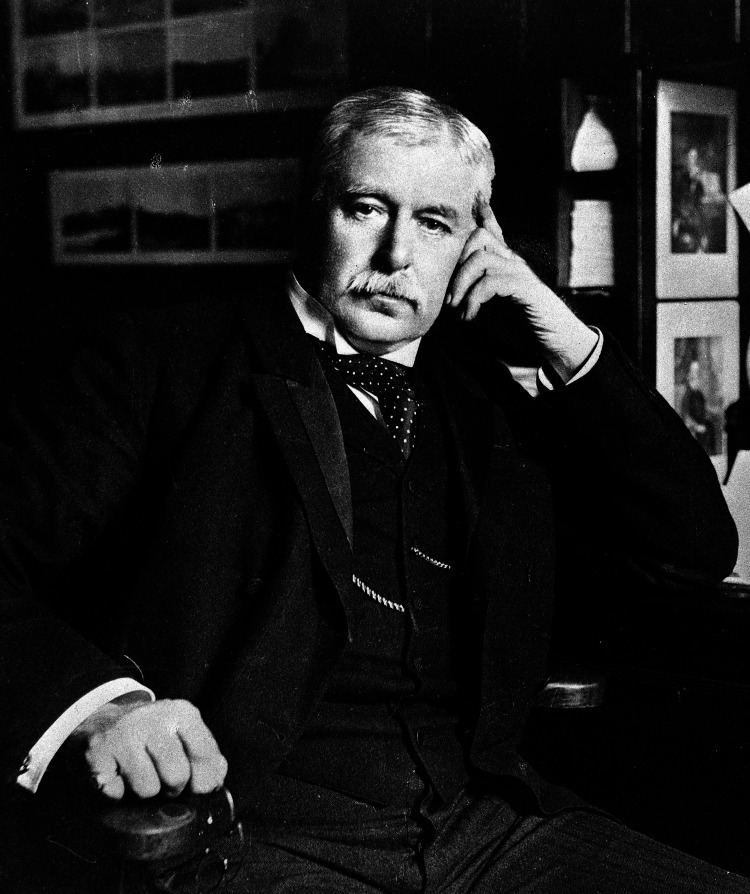
Sir Patrick Manson Reference: [2]

Born on October 3, 1844, in Aberdeenshire, Scotland, Patrick Manson (Figure 1) was the eldest child of John Manson, a bank manager, and Elizabeth Livingston. From a young age, Manson showed a strong interest in natural history, particularly in the study of insects. He pursued his education at the University of Aberdeen, where he earned his M.B. degree in 1865 at the age of 21, followed by his M.D. a year later. In his early stages, he rendered medical service for the Chinese Imperial Maritime Customs, first in Takao, Formosa, and later at the Amoy port in China [1]. During this period, Patrick Manson's foundational passion for natural history, particularly entomology, was pivotal in his groundbreaking advancements in parasitology.

Service unto the tropics

Inspired by his brother, Manson took a position as a medical officer in the Customs Service of Takao, Formosa (modern-day Kaohsiung, Taiwan), in 1866. Apart from his primary responsibility as a port medical officer, he worked in a missionary hospital gaining invaluable experience with a wide range of diseases, from filariasis to leprosy, without any formal supervision. Equipped only with his clinical and observational skills and meticulous record-keeping, Manson handled unique medical challenges. He was well known for inculcating novel medical techniques to treat his patients, and his keen observation skills led him to derive causal hypotheses for many ailments [3]. He also gained popularity among Chinese inmates who were initially reluctant but then preferred Western medical procedures on seeing Manson's translucency in treating his patients [4]. Thus, Patrick Manson significantly impacted local health systems through his public health reforms.

Notable discoveries

Patrick Manson's deep-seated interest in insects and exceptional observational skills enabled him to investigate the transmission pathways of tropical diseases such as filariasis. Additionally, his early focus on the life cycles of microscopic organisms and their ecological interactions informed his later research on trematodes and malaria transmission, cementing his legacy as a pioneer in tropical medicine and parasitology.

Filariasis

During his study leave in London, Manson was keen to seek the latest knowledge on tropical diseases and entomology. But he was disheartened by the lack of organized, up-to-date information. However, he came across Timothy Lewis's work from the library of the British Museum, which described the discovery of a worm, a nematode named* Filaria sanguinis hominis *(later identified as *Wuchereria bancrofti*) [5], from the blood and chylous urine of a patient with chyluria. This caught Manson's attention, as he had treated numerous elephantiasis patients who also had chyluria. Upon returning to Amoy, he began investigating this connection further.

Manson began his clinical experiments on filariasis-infected individuals. Fifteen (7.9%) out of 190 Chinese individuals with microfilaremia showed signs of elephantiasis or other health issues like recurrent fever and edema. After analyzing 670 patients, he found that one in 10.8 people in Amoy was infected, with the incidence rate increasing to 33.3% in the elderly. Manson's sharp observational skills led him to discover that microfilariae concentrations in the blood peaked around midnight, aligning with both human sleep cycles and mosquito-biting behavior. His groundbreaking findings were published in the Journal of the Linnean Society of London, Zoology in 1878 [6].

While examining the blood of elephantiasis patients, Manson also discovered that sheathed microfilariae remained unchanged at body temperature but shed their sheaths at room temperatures in tropical climates. He tracked the metamorphosis of microfilariae in the alimentary tract of *Culex fatigans *(now known as* Culex quinquefasciatus*) mosquitoes on feeding infected blood [6]. Through multiple mosquito dissections, Manson found that these microfilariae moved from the mosquito's digestive tract to its thoracic muscles for further development. Following this in 1899, Thomas Bancroft finally demonstrated that infected female mosquitoes could transmit the parasite back to humans through biting [7]. Thus, Manson's research paved the way for the discovery that mosquitoes serve as vectors for human-to-human transmission.

Collaboration With Ronald Ross: Malaria Research

In 1892, while in London, Manson first encountered information on malarial parasites and developed his "mosquito theory" of malaria transmission. He had long observed the exflagellation of the malarial parasite (later identified as male gametocytes) in blood smears from infected patients. Building on his earlier success in uncovering the mosquito-borne transmission of filarial worms, Manson applied these insights to malaria, a similarly devastating tropical disease. One of his most renowned collaborations was with Sir Ronald Ross, a British physician who later won the Nobel Prize in Medicine for his groundbreaking work on malaria. Manson encouraged Ross to investigate the possibility of mosquitoes playing a role in malaria transmission. In 1894, Manson wrote a pivotal letter to Ross, urging him to seek out the mosquito in the life cycle of the malarial parasite. In 1897, after several failed experiments and considerable persistence, Ross made his breakthrough discovery. He found malarial parasites (now known as *Plasmodium*) in the stomach of *Anopheles* mosquitoes that had fed on malaria-infected birds. He observed the growth of the parasites, which were similar to what Manson had predicted. Together, Manson's theoretical foundations and Ross's experimental findings greatly advanced the understanding of malaria transmission [8].

Other Tropical Diseases

Manson later identified the lung fluke *Paragonimus westermani *(formerly *Distoma ringeri*) by observing large operculated eggs in the sputum of a patient who had succumbed to endemic hemoptysis. He hypothesized that these flukes required an intermediate host, typically a freshwater crustacean like snails, to complete its life cycle. This theory was confirmed in 1916 by Nakagawa [9]. Manson's work on freshwater snails as vectors was later extended to schistosomiasis. His contributions to tropical diseases were extensive, including the discovery of *Filaria diurna* (now known as *Loa loa*), *Filaria demarquaii* (now known as *Mansonella ozzardi*), *Filaria perstans* (now known as *Mansonella perstans*) [10], the tapeworm *Bothriocephalus mansoni *(now known as *Spirometra* *mansonoides*), *Schistosoma mansoni,* and the dermatophyte *Trichophyton concentricum* (causing tinea imbricata). Even the mosquito genus *Mansonia *was given in honor of his scientific contribution [11-13].

Contribution to medical education

Manson relocated to the Civil Hospital for Europeans in Hong Kong after 16 years of medical service in Takao and Amoy. There he co-founded the Alice Memorial Hospital along with Sir Kai Ho Kai, supported by the London Missionary Society. It is also the teaching hospital for the Hong Kong College of Medicine for Chinese, which later evolved into the University of Hong Kong (HKU). In addition to establishing the first Western hospital and medical school in Hong Kong, Manson also established the Hong Kong Medical Society in 1886. While addressing the society members, Manson expressed his concern about the lack of in-depth knowledge in tropical medicine, which often led to misdiagnosis.

Upon his return to London in 1889, Patrick Manson was determined to address the lack of specialized education in tropical diseases. His vision for creating an institution dedicated to tropical medicine, however, was not without its hurdles. Several challenges emerged, ranging from institutional resistance to funding difficulties and skepticism from the medical community. Despite Manson's reputation as a pioneering parasitologist, the reluctance of established medical bodies to support his vision made fundraising an uphill battle. Gaining approval from licensing bodies to offer specialized tropical medicine qualifications involved navigating layers of red tape. The idea of tropical medicine as a legitimate field worthy of certification was novel, and it took Manson considerable time and effort to convince these bodies of the necessity of such a school. Manson eventually managed to garner the support of the Colonial Office by pointing to the high rates of disease-related deaths among British soldiers and colonial administrators in tropical regions. He argued that specialized training in tropical medicine was crucial for improving the health of British personnel abroad. After years of persistence, Manson finally succeeded in establishing the London School of Hygiene and Tropical Medicine in 1899. The school was initially affiliated with the University of London, offering specialized training for physicians who would be deployed to tropical regions. Even at an older age, Manson traveled up to 9 miles several times a week to teach at the school. Like many eminent scientists, he remained deeply invested in fostering the next generation's interest in patient care and advancing the control of infectious diseases.

Public health initiatives

As a seasoned clinician-scientist, Manson firmly believed in the principle that "prevention is better than cure." During his stay in Amoy, he promoted smallpox vaccination which significantly reduced both the incidence and death rates of the disease. While in Hong Kong, Manson and his colleagues observed that the British garrison faced an annual death rate of 3-13.6%, primarily due to infectious diseases. In response, they advocated for improved sanitation and preventive measures to address the situation.

In 1886, they established a dairy farm in the Pokfulam district for hygienic and affordable provision of dairy products for pregnant women, children, and patients. Manson's research was the basis for several modern clinical guidelines and epidemiological programs in tropical medicine, emphasizing the importance of public health initiatives and preventive care in combating infectious diseases. His works still resonated after his lifetime in the public health realm, namely, the global campaign to eliminate lymphatic filariasis by the World Health Organization in 2000 [14], a movement that was built upon the strong foundation laid by Manson.

## Conclusions

Manson passed away in 1922, leaving behind a legacy of exceptional clinical practice and pioneering research even without any formal mentorship. His extensive time spent in tropical regions posed significant health risks, yet he exemplified excellence in clinical work, medical education, and microbiological research. His research contributions underscored the importance of vector control in preventing tropical disease transmission, thus earning him the title "father of tropical medicine." He speculated that various mosquito species could influence the malaria parasite, suggesting that differing levels of virulence might depend on the specific mosquito species acting as the parasite's alternative host. These insights were early precursors to the modern concepts of vector capacity and vector competence. Manson believed that many errors in medicine stem not from a lack of knowledge but from a failure to observe. He asserted that it is not the microscope itself but the observer's insight that matters. He also advised against ignoring what one may not wish to see, as such observations could often lead to significant discoveries.

## References

[1] Manson-Bahr PH, Alcock A (1927). The life and work of Sir Patrick Manson. https://books.google.com.ph/books/about/The_Life_and_Work_of_Sir_Patrick_Manson.html?id=IidDAAAAYAAJ&hl=en&output=html_text&redir_esc=y.

[2] (2024). File:Patrick Manson3.jpg. https://commons.wikimedia.org/w/index.php?curid=33185828.

[3] Manson P (1892). Remarks on an operation for abscess of the liver. Br Med J.

[4] To KK, Yuen KY (2012). In memory of Patrick Manson, founding father of tropical medicine and the discovery of vector-borne infections. Emerg Microbes Infect.

[5] Lewis T (1877). Filaria sanguinis hominis (mature form), found in a blood-clot in nævoid elephantiasis of the scrotum. Lancet.

[6] Manson P (1878). On the development of Filaria sanguinis hominis, and on the mosquito considered as a nurse. Zool J Linn Soc.

[7] Mackerras IM, Marks EN (1973). The Bancrofts: a century of scientific endeavour. Proceedings of the Royal Society of Queensland.

[8] Ross R (1923). Memoirs, with a full account of the great malaria problem and its solution. https://search.worldcat.org/title/1048808925.

[9] Nakagawa K (1917). Human pulmonary distomiasis caused by Paragonimus westermanni. J Exp Med.

[10] Haynes DM (2000). Framing tropical disease in London: Patrick Manson, Filaria perstans, and the Uganda sleeping sickness epidemic, 1891-1902. Soc Hist Med.

[11] (2000). Medicine in the twentieth century.

[12] Manson P (1902). Report of a case of bilharzia from the West Indies. Br Med J.

[13] Castellani A (1913). Note on the ætiology of some tropical dermatomycoses (Tinea cruris, Tinea flava et nigra, Tinea imbricata). Proc R Soc Med.

[14] (2024). Global Programme to Eliminate Lymphatic Filariasis. https://www.who.int/teams/control-of-neglected-tropical-diseases/lymphatic-filariasis/global-programme-to-eliminate-lymphatic-filariasis.

